# Thinking out of the box—by thinking in other boxes: a systematic review of interventions in early entrepreneurship vs. STEM education research

**DOI:** 10.1007/s11301-021-00248-3

**Published:** 2021-11-30

**Authors:** Joanna Barth, Katrin Muehlfeld

**Affiliations:** Management, Organization Studies and Human Resource Management, Trier University, Universitätsring 15, 54296 Trier, Germany

**Keywords:** Entrepreneurship education, Entrepreneurial self-efficacy, Cross-disciplinary research, Primary school, Secondary school, STEM, A21, C90, I20, J24, L26

## Abstract

Scholars increasingly converge on the view that entrepreneurship education (EE) should start early during the formative years of individuals’ educational careers, ideally in primary and secondary education. They also agree that promotion of attitudinal factors, especially, entrepreneurial self-efficacy (ESE) is of crucial importance. Yet, empirical evidence on effective interventions to foster ESE in early EE is still scarce. Therefore, this study, first, systematically reviews and systematizes this limited literature consisting of eight quantitative studies. Second, in order to develop suggestions for future ESE-oriented interventions in early entrepreneurship education, the study draws on insights from systematically reviewing a second, related yet already more developed literature: research on self-efficacy-oriented interventions in early STEM (science, technology, engineering, and mathematics) education. Third, this study compares the interventions across both research streams in terms of research design, theoretical framework, structure and content of the interventions, and the findings of the studies. As a result, it derives implications for future research on ESE-oriented interventions in early EE: regarding the research design (e.g., use of several treatments); the structure and content of the programs like pedagogical and methodological components (e.g., use of specific learning strategies); analysis of potentially important moderating variables (e.g., gender, social background). Finally, the study discusses the potential for developing cross-disciplinary interventions aimed at simultaneously encouraging self-efficacy in the domains of STEM and entrepreneurship.

## Introduction

Occupationally-related attitudes and mindsets already develop during childhood and adolescence (Peterman and Kennedy 2003; Wilson et al. 2004). Preparing individuals for the labor market of the future thus starts at school. But what are the demands of this future labor market? Scholars broadly agree that work arrangements outside of standard employment relationships are becoming increasingly pervasive, necessitating growing numbers of people to be able to effectively engage in behaviors commonly associated with entrepreneurship (Frese et al. 2014)—and that educational efforts to prepare them accordingly should start already during childhood and early adolescence.

Yet, sole promotion of *cognitive* entrepreneurial skills (e.g., knowledge, competencies) appears to be insufficient. Nurturing, as a complementary resource, *non-cognitive* skills such as, in particular, a positive self-efficacy concerning challenges associated with entrepreneurship seems necessary (Grossman and Porche 2014; Newman et al. 2019). Self-efficacy, in general, describes the assessment of one's abilities to cope with new or difficult situations (Bandura 1977). It is flexible and malleable in response to experiences (Bandura 1997; Usher 2009). In addition to a general self-efficacy construct, domain-specific self-efficacies exist as well (Bandura 1997; Newman et al. 2019), such as entrepreneurial self-efficacy (ESE). ESE refers to an individual's belief in his or her capability to perform tasks and roles aimed at entrepreneurial outcomes (Chen et al. 1998). As such, it constitutes a particularly fundamental outcome variable that (early) entrepreneurship education (EE) may target: First, it represents a key antecedent to other important outcome variables such as, for example, entrepreneurial intention. Conceptually, this relationship is based on Ajzen’s (1985, 1991) theory of planned behavior—one of the most influential theoretical frameworks capturing fundamental links between motivation, intention, and behavior in the entrepreneurial domain and beyond. Its relevance as such and its importance for these “downstream” variables have also been documented both by a vast number of empirical studies (Bagheri and Pihie 2014; Wilson et al. 2009; Zellweger et al. 2011). However, second, the particular relevance of ESE as a target outcome variable of (early) EE derives from at least one further reason: its broader importance as an entrepreneurially-oriented non-cognitive skill that will be sought after in workers even if they do not ‘take the plunge’ (e.g., European Communities 2007; Frey and Osborne 2017; Newman et al. 2019). ESE appears to be an essential resource of workers in the future labor market (Newman et al. 2019), whether or not they become ‘entrepreneurs’ in a traditional sense, rendering it a particularly important outcome variable of early entrepreneurially-oriented educational efforts.

Yet, this importance of ESE from both a directly entrepreneurial as well as a generalized labor market perspective stands in contrast to the relative dearth of scholarly insights into how to foster it during the formative years of primary and secondary education. The nascent literature within EE research (Rodrigues et al. 2012) that examines the promotion of ESE in primary and secondary education is still scant: In a structured literature review considering outcomes of entrepreneurial education in primary and secondary schools, in general, Brüne and Lutz (2020) identified no more than 21 qualitative and quantitative studies, in total. In order to offer a comprehensive overview of consequences-focused EE research, they included a wide range of outcome variables (e.g., entrepreneurial skills, intention, self-efficacy) and a broad definition of EE, referring to Leffler and Svedberg (2005) as including both specific programs and entrepreneurial contexts and entrepreneurial situations at school. Their review is particularly valuable in offering a broad perspective, which allows for uncovering differences and similarities in impact exerted by the different measures across various outcome variables, including considerations of moderating variables such as gender, age, or role models (Brüne and Lutz 2020). Moreover, their approach illustrates the remarkable variety of measures adopted as part of entrepreneurship programs in schools. Yet, at the same time, it implies that an in-depth analysis of the actual designs of the interventions is beyond the scope of their study. Consequently, their review points to an important gap in the literature: a systematic review and analysis focusing specifically on the intervention designs of EE programs in primary and secondary schools.

The present literature review addresses this gap by zooming in not only on ESE as a core target variable of early EE but also by specifically analyzing intervention designs aimed at fostering it. Breslin and Gatrell (2020) recently proposed the ‘miner-prospector continuum’ as a useful metaphor for understanding distinct approaches to building insights from literature reviews. Specifically, they suggested that literature reviews may be positioned on a continuum ranging from strictly disciplinary (more ‘miner-oriented’) to fundamentally cross-disciplinary (more ‘prospector-oriented’) approaches, which seek to introduce novel perspectives from outside of established domains of study. In the context of the present study, we consider a cross-disciplinary, ‘prospector-oriented’ approach as particularly promising: The literature dedicated specifically to analyzing how entrepreneurially-oriented interventions in primary and secondary schools may foster ESE is still in its infancy. This implies that there are, to date, relatively few studies to draw upon when adopting a ‘miner-oriented’ approach (Brüne and Lutz 2020). Additionally, the importance of ESE from a generalized labor market and, thus, from a broader educational perspective, suggests that a more comprehensive approach, linking ESE-oriented early EE with other educational disciplines, may yield valuable insights into educational ‘best practices’ in terms of fostering self-efficacy, more generally. In sum, drawing additionally from other, already more developed educational research fields promises to facilitate gaining novel insights that enrich the discussion of interventional options in early EE. Ultimately, such an approach should allow us to derive more specific implications for both future research and practice aimed at designing effective entrepreneurship interventions in schools.

But which field to draw upon? Here, we propose that research on self-efficacy-oriented interventions in early STEM education (science, technology, engineering, and mathematics) has several advantages as a complement to research on ESE-oriented interventions: First, the analysis of self-efficacy-oriented interventions in early STEM education is much more advanced, thus offering a glimpse into the future regarding which avenues may be more or less promising for EE research to pursue (e.g., Moos and Azevedo 2009). Second, it allows for explicitly considering possible cross-disciplinary linkages between EE and an educational perspective aimed at addressing the second major trend affecting the labor market of the future: Increasing automation and digitalization (Frey and Osbourne 2017) imply that the importance of STEM-related skills is on the rise, and with it the need for educational institutions such as schools to promote STEM-related self-efficacies, both domain-specific as well as generalized STEM-related—especially in conjunction with entrepreneurial skills. Indeed, the rising relevance of STEM-related skills in the labor market of the future is concomitant with an increasingly prominent role of entrepreneurial skills both outside of and within standard employment relationships, where it is often discussed as intrapreneurship or corporate entrepreneurship (Corbett et al. 2013). Moreover, STEM sectors represent the most promising industries for entrepreneurial endeavors, with technologies such as artificial intelligence (AI) and robotics increasingly permeating all areas of life (Atkinson and Mayo 2010; Csorny 2013). Third, both STEM subjects (e.g., Shin et al. 2016) and EE are often perceived as particularly challenging for learners, rendering it particularly difficult to foster domain-specific self-efficacy among students. Thus, successful STEM interventions may be a basis for the derivation of implications for EE, especially concerning type and structure of the tasks or the collaboration between students.

As a complement to these literature-based arguments, we conducted a small-scale, two-part primary data collection: first, an illustrative survey among university^1^ students (*N* = 135, with 59.3% being females; average age = 23 years, SD = 2.886); and, second, qualitative interviews with teachers. In the survey, we asked students (1) to retrospectively assess their primary and secondary education regarding a variety of subjects, and (2) to prospectively gauge the relevance of both STEM education and entrepreneurially-oriented education for the labor market. Results from this survey suggest that indeed students considered STEM and entrepreneurial skills as vital for the labor market of the future: 48.9 percent of respondents viewed digital literacy (on a scale from (1) “not at all important” to (5) “very important”) as “very important” (mean = 4.33, SD = 0.792), followed by entrepreneurial skills (mean = 3.68, SD = 0.911) and general STEM-related skills (mean = 3.68, SD = 0.951). Further, when asked to classify learning for different (school) subjects according to difficulty, 35.6 percent of respondents considered STEM learning as the most difficult (ranging from (1) “most difficult” to (6) “least difficult”), with entrepreneurship being the second most difficult subject based on a comparison of mean values (mean = 3.12, SD 1.506). In the interview study, we identified possible reasons for these results: Both areas appear to be perceived by many students as comparatively abstract (with example quotes by teachers such as, e.g., “natural sciences are very abstract and therefore challenging subjects that are, depending on the target group, difficult to teach”,^2^ STEM teacher at a community school in Germany; “students may be able to cope when the lessons draw on examples of companies that are close to their everyday life experiences, (…) otherwise some things are difficult to explain, too abstract”,^3^ entrepreneurship teacher at a vocational school in Germany). Overall, this anecdotal evidence tentatively supports the notion that both disciplines seem to face similar challenges in primary and secondary education. In sum, research on STEM education aimed at fostering self-efficacy appears to constitute a particularly suitable starting point for engaging in a cross-disciplinary discourse regarding the advancement of ESE-oriented early EE. Thus, this study seeks to identify key implications for the design of future interventions in primary and secondary EE education aiming at promoting ESE.

To this end, we first provide a systematic review of research in EE, which has addressed the promotion of ESE in primary and secondary education through interventions. We compare the experiments and quasi-experiments that comprise this nascent literature based on four different criteria: (1) research design, (2) theoretical framework of the papers, (3) structure and content of the interventions themselves, and (4) the findings of the studies. Based on this review, we identify several unaddressed areas of research questions, the analysis of which we propose may benefit from turning towards STEM education research. In a second step, using the same set of criteria, we therefore review corresponding studies in STEM education research that analyze the effectiveness of interventions in primary and secondary education aimed at promoting STEM-related self-efficacies. Third, we consider the results from both review parts in conjunction, and, specifically, ask what research on EE stands to gain from taking into account insights from this literature on STEM education. This cross-disciplinary perspective also enables us to derive novel implications for the design of future interventions geared at developing ESE in early EE.

## Promoting entrepreneurial self-efficacy (ESE) in primary and secondary education

### Methods

**Fig. 1:**
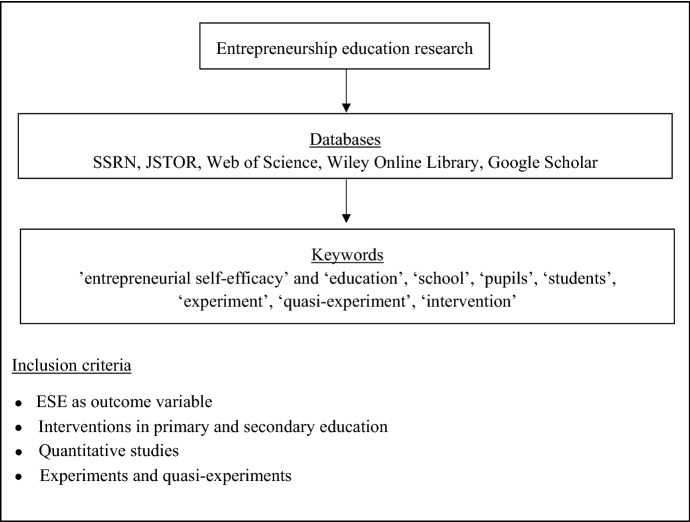
Review approach. Please note that additional illustrations of the results of this literature review (tables and figures) are available from a complementary online appendix at https://osf.io/z5nsc/?view_only=2c62b185a1474433b9c8cee766aee7f8

Based on recommendations by Fisch and Block (2018) and in line with best practices (Short 2009), we used Web of Science, Google Scholar, JSTOR, and related databases to identify studies that have investigated the promotion of ESE by means of interventions in primary and secondary education. First, we integrated the keyword ‘entrepreneurial self-efficacy’ in different combinations with terms like ‘school’, ‘pupils’, ‘students’, ‘education’, ‘experiment’, and/or ‘quasi-experiment’. In a second step, we used backward and forward searches based on the citations of these articles (Levy and Ellis 2006). Overall, we found 195 studies. First, to ensure a high quality of the results, we only included peer-reviewed journal papers and conference papers (112 papers). Second, we included only studies that fulfilled the following criteria: (1) Since it is a central aim of this study to examine the effectiveness of interventions on the development of ESE, we only considered studies that included ESE as a dependent variable. (2) In line with our research focus, we included only studies that investigated interventions during primary and secondary education. (3) Further, we restricted the review to quantitative studies, and, specifically, to experimental and quasi-experimental designs, which allow, at least in principle and if well designed, for gauging causal relationships (e.g., Köhler et al. 2017; Slavin 2002) also in pedagogical contexts.^4^ According to Shadish (2002), experiments are studies in which an intervention is carried out to observe its effects. In contrast to experiments in which the groups are randomly assigned to different conditions, in quasi-experiments, the assignment is not random. Figure 1 shows an overview of our approach. Overall, we identified eight studies that fulfilled the criteria. The earliest study was published in 2011, the other ones in 2013, 2014, 2017, 2018, 2019, and 2020; illustrating that this is a literature still in its infancy but attracting growing interest from scholars, in line with the increasing relevance of the topic itself.

### Literature review: entrepreneurship education research

#### Results of comparing research designs

We compared the studies with respect to (i) whether or not development of the focal intervention had been an *integral part of the study*, (ii) the *number of programs covered* by study, (iii) the use of either an *experimental* or a *quasi-experimental design*, and (iv) whether or not they offered a *comparison across multiple, different interventions*^5^.

First, seven studies assessed the impact of interventions that had been developed and implemented independent from and for purposes other than those of the studies themselves, thus did not represent an *integral part of the study* itself. Sánchez (2013) did not provide any information as to when and by whom the focal program (‘E Vitamin’) was developed.

Second, in terms of *number of programs covered*, seven studies investigated the outcomes of a single such program. Bux and van Vuuren (2019) investigated the effects of two variations within the same program—Junior Achievement South Africa (JASA) entrepreneurship education intervention—which differed by duration (‘short’ vs. ‘long’). Volery and colleagues (2013) included three different programs and motivated the joint inclusion based on the notion that this approach would allow them to cover all major Swiss entrepreneurship programs at the upper-secondary level, including vocational, technical, and commercial schools. Moreover, they noted that all three programs shared common features in terms of content and pedagogical elements in that they were designed to offer basic entrepreneurial knowledge (e.g., product development, marketing, finance, business planning) and entrepreneurial soft skills (e.g., creative thinking, new product development) and took into account similar educational strategies such as action learning (e.g., field trips, development of business ideas, consultation with practitioners). As the programs also differed in various potentially important respects at the same time (e.g., overall duration and timing of the sessions varied from intensive one-week courses to semester-long courses), a cross-program comparison would have been interesting, too, but was presumably beyond the scope of the study, due to sample size requirements. A common feature across all evaluated programs in the eight studies was that, at the school level, all programs were offered on a voluntary basis. Thus, none of the programs was part of the compulsory curriculum in any of the educational contexts and countries covered. If a school decided to participate, however, all affected students had to participate, with two exceptions: Volery and colleagues (2013) integrated, in addition to compulsory programs, a program with voluntary participation at the student level. Sánchez (2013) analyzed a program that was offered in schools as a free elective subject.

Third, regarding the use of *experimental* or *quasi-experimental designs*, two studies used experiments (Bergman et al. 2001; Rosendahl Huber et al. 2014), five studies employed a quasi-experimental approach (Sánchez 2013; Streicher et al. 2019; Volery et al. 2013). One study (Cárcamo-Solís et al. 2017) could not be assigned due to a lack of information. Further, six studies used a pre-posttest design with one measurement before the intervention and one immediately after the intervention. One study (Bux and van Vuuren 2019) did not use a pretest. Thus, it is difficult to determine whether the observed effects were triggered by the interventions, implying that the research design does not fully adhere to the requirements of the experimental approach. Only one study (Volery et al. 2013) made use of a pre-, post-, and post-posttest design. Six studies used a classic control group design with one experimental and one control group. The studies by Cárcamo-Solís and colleagues (2017) and Bux and van Vuuren (2019) did not use a control group. Bux and van Vuuren (2019) investigated two versions within the same program (distinct in terms of duration: ‘short’ vs. ‘long’), thus, using two experimental groups.

Finally, fourth, we compared the studies based on whether they offered a *comparison across multiple, different interventions*. Five studies assessed the effects of a single intervention in a single country context (Israel: Bergman et al. 2011; Mexico: Cárcamo-Solís et al. 2017; Germany: Grewe and Brahm 2020; Netherlands: Rosendahl Huber et al. 2014; Spain: Sánchez 2013). Streicher and colleagues (2019) evaluated the effects of a single program, which was, however, implemented in several countries, i.e., Slovenia, Austria, Portugal, and Luxembourg. Volery and colleagues (2013) and Bux and van Vuuren (2019), in turn, focused on an assessment within a single country context (South Africa: Bux and van Vuuren 2019; Switzerland: Volery et al. 2013) but included either several entrepreneurship programs (Volery et al. 2013) or several versions of the same program (Bux and van Vuren 2019: ‘short’ vs. ‘long’ version). Volery and colleagues (2013) analyzed three different entrepreneurship programs used in Switzerland at the upper secondary level, considering thus three distinct interventions: (1) one offered by the Swiss Federal Office for Professional Education and Technology, (2) the 'Company Program' of Youth Enterprise Switzerland (a member of Junior Achievement Worldwide) and (3) the 'Learn to undertake' program. They also highlighted similarities and differences in the design of the three programs. Table 1 offers an overview of the research designs used in EE and STEM research.

**Table 1:** Overview of the research designs used in entrepreneurship and STEM education research

Research field	Author(s) (year)	Exp./Quasi-Exp	Measurement	Group design	Country
EE	Bergman et al. (2011)	E	Pre/Post	T/C	Israel
	Bux and van Vuuren (2019)	QE	Post	2T	South Africa
	Cárcamo-Solís et al. (2017)	n.a	Pre/Post	T	Mexico
	Grewe and Brahm (2020)	QE	Pre/Post	T/C	Germany
	Rosendahl Huber et al. (2014)	E	Pre/Post	T/C	Netherlands
	Sánchez (2013)	QE	Pre/Post	T/C	Spain
	Streicher et al. (2019)	QE	Pre/Post	T/C	Slovenia, Austria, Portugal, Luxembourg
	Volery et al. (2013)	QE	Pre/Post/Post	T/C	Switzerland
STEM	Falco et al.(2010)	QE	Pre/Post/ Post	T/C	n.a
	Falco and Summers (2017)	E	Pre/Post	T/C	USA
	Feldhausen et al. (2018)	QE	Pre/Post	2T	USA
	Feng and Tuan (2005)	QE	Pre/Post	T/C	Taiwan
	Hiller and Kitsantas (2014)	QE	Pre/Post	T/C	USA
	Huang et al. (2018)	QE	Post	1T	USA
	Isiksal and Askar (2005)	E	Pre/Post	2T/1C	Turkey
	Kitsantas et al. (2004)	E	Pre/Post	8T	n.a
	Leonard et al. (2016)	QE	Pre/Post	3T	USA
	Plant et al. (2009)	E	Post	6T	USA
	Rakoczy et al. (2019)	RFT	Pre/Post	T/C	Germany
	Ramdass and Zimmerman (2008)	E	Pre/Post	T/C	USA
	Samsudin et al. (2020)	QE	Pre/Post	T/C	Malaysia
	Schunk (1985)	E	Pre/Post	2T/1C	USA
	Schunk and Cox (1986)	E	Pre/Post	8T/1C	USA
	Star et al. (2014)	E	Pre/Post	3T	USA
	Weisgram and Bigler (2007)	E	Pre/Post	T/C	n.a

The table offers an overview of the research designs used in EE and STEM education research, with a focus on the different types of experimental designs (experiments versus quasi-experiments versus randomized field trial), times of measurements (Pre = before the intervention; Post = immediately after the intervention, Post/Post = immediately after the intervention and long-term after the intervention), the design of the treatment conditions (T = design only includes one or more treatment condition, T/C = design includes one or more treatment conditions and a control group), and the country, where the intervention took place

#### Results of comparing the underlying theoretical frameworks

The studies differ considerably with respect to the underlying theoretical frameworks. Four studies (Bergman et al. 2011; Cárcamo-Solís et al. 2017; Grewe and Brahm 2020; Rosendahl Huber et al. 2014) did not explicitly refer to any specific theoretical framework but instead, in their literature reviews, focused on describing the empirical findings of prior research on promoting ESE by means of EE and/or on implicitly drawing upon prior conceptualizations.

In turn, Streicher and colleagues (2019) and Sánchez (2013) derived their hypotheses based on the theory of planned behavior (TPB) by Ajzen (1985, 1991), in which self-efficacy represents a starting point of cognitive processes. According to TPB, the intention aimed at engaging in any kind of behavior arises from three sources: (1) attitudes towards the behavior, (2) subjective norms, which refers to the perceived social pressure to perform a behavior, and (3) perceived behavioral control; with perceived behavioral control being closely related to Bandura’s concept of self-efficacy. Both studies—Streicher et al. (2019) and Sánchez (2013)—each combined this theoretical framework with complementary theoretical perspectives. Sánchez (2013) additionally drew on human capital theory (Becker 1964). Streicher et al. (2019) complemented TPB with the choice model of the social cognitive career theory (SCCT) (Lent et al. 1994). SCCT focuses on career choice decisions and conceives of self-efficacy as the result of a learning experience and, at the same time, as antecedent to factors that influence professional outcomes, such as outcome expectations (Lent et al. 2000), interests, choice goals, choice action (Zhao et al. 2005) and the attribution of success or failure during the occupational choice process. In addition, it explicitly differentiates between personal (e.g., gender, ethnicity) and environmental (certain role models or the financial situation) factors, which also influence the career choice directly or indirectly. Volery and colleagues (2013) also drew on human capital theory, akin to Sánchez (2013), but did not link it to any other theory.

Bux and van Vuuren (2019) briefly referred to Bandura’s Social Cognitive Theory (SCT) (1997) and also pointed to a need to complement it with a suitable pedagogical perspective in the context of EE, but did not elaborate in detail on these considerations, nor did they clearly base their hypotheses derivation on any of these conceptual frameworks, instead, implicitly, they seemed to draw, primarily, on TPB. Overall, thus, only three studies explicitly specified the theoretical perspective(s) underlying their conceptual frameworks and hypotheses derivation, with TPB representing the most frequently employed theory. Moreover, none of the studies, including those that explicitly specified a theoretical perspective as their foundation, referred to didactic or pedagogical theories—presumably, because they all focused on *assessing* the effects of the focal programs and were not concerned with or involved in the actual design of interventions.

#### Results of comparing the structure and content of the interventions

Next, we examined the structure of the programs—especially with regard to (i) the *target group*, (ii) the *duration of the interventions*, (iii) the *type and structure of the tasks* given to participants of the programs, and (iv) the *specific skills* that the focal program sought to promote.

First, *target groups* can be characterized based on *participants’ age* and the *specification of the program to different class levels*. Across all studies, mean participant age varied between eleven and 20 years. As the interventions were designed for implementation in schools, programs were often described according to class levels, ranging from fifth to final grade. Only one study addressed a specific grade level (Bergman et al. 2011). Four further studies (Bux and van Vuuren 2019; Cárcamo-Solís et al. 2017; Grewe and Brahm 2020; Volery et al. 2013) considered programs designed for several grade levels. The remaining three studies (Rosendahl Huber et al. 2014; Sánchez 2013; Streicher et al. 2019) did not provide any specific information about cross-year use of the focal interventions. In terms of types of schools, two studies examined interventions at elementary schools (Cárcamo-Solís et al. 2017; Rosendahl Huber et al. 2014), one at a middle school (Sánchez 2013), and five at high schools (Bergman et al. 2011; Bux and van Vuuren 2019; Grewe and Brahm 2020; Streicher et al. 2019; Volery et al. 2013).

Second, considering the *duration of the interventions,* five interventions lasted more than 10 days, specifically between 6 months and a year (Bergman et al. 2011; Grewe and Brahm 2020; Sánchez 2013; Streicher et al. 2019; Volery et al. 2013). Rosendahl Huber and colleagues (2014) examined a program with a duration of in total 5 days, stretched out over the course of between two and four weeks. Two studies (Cárcamo-Solís et al. 2017; Bux and van Vuuren 2019) did not provide explicit information regarding the intervention’s duration, but Bux and van Vuuren (2019) described two versions of the intervention as ‘short’ and ‘long’. Regarding the duration of individual sessions within each of the interventions, none of the studies provided explicit information. Overall, the interventions were more long-rather than short-term oriented.

Third, regarding *type and structure of the tasks* given to students, six studies used an action-oriented approach. That is, the programs focused on aspects like ‘learning by doing’ or, in this context, ‘learning by founding’. In contrast to a more teacher-centered approach, in which learning is pre-structured, guided, and evaluated by the teacher, this approach is more learner-centered: students encounter problems and solve them independently (Wright 2011). Teachers primarily act as learning companions and supporters, while students themselves are responsible for organizing their learning. One of these more action-oriented teaching models was the Mexican program ‘My first company: Entrepreneurship by playing for primary school children’ (Cárcamo-Solís et al. 2017). The students founded a fictitious mini-company, took over various business positions, such as a financial manager or a product manager, and presented their entrepreneurial ideas to stakeholders (e.g., family or classmates). Another approach with a focus on ‘learning by being an entrepreneur’ was the JUNIOR program (Grewe and Brahm 2020). With more than 28.000 mini-companies in 2018, this program is one of the most frequently used ones in Europe (Oosterbeek et al. 2010), with a similar focus to ‘My first company’. Accompanied by a teacher and a business mentor, students develop an idea, found and organize an own company, acquis seed capital, execute and administrate the production of their goods, organize their marketing and sales, and finally close their company. Another action-oriented program was ‘BizWorld’ (Rosendahl Huber et al. 2014), the basic structure of which is also similar to that of ‘My first company’. ‘BizWorld’ is one of the most well-known and internationally established programs for early EE, aimed at students aged 11–12 years old. It originated in the U.S. in the late 1990s and meanwhile more than 350,000 children from more than 80 countries have participated (Rosendahl Huber et al. 2014). Teams of five to six students are usually grouped together to act as a founding team of a fictitious company. Ultimately, at the end of the course, the teams’ performance is assessed at class/school level, and the winners are rewarded with a small gift and an official winning certificate. The program ‘Young Entrepreneurs – Israel’ (Bergman et al. 2011) has a similar overall approach but a stronger emphasis on competition. Here, each mini-company is supported by an experienced businessperson, a school teacher, and a trainer (a student in relevant disciplinary field). At the end of the year, the mini-companies compete with other mini-companies in a regional, national, and international competition. The Spanish program ‘E-Vitamin’ assessed by Sánchez (2013) includes ‘acting like an entrepreneur’ (e.g., by learning about accounting, finance, marketing, and by writing a business plan) as well as the establishment of explicit links with entrepreneurial business practice (e.g., through discussions with entrepreneurs and networking events). It uses different methodologies, like discussion of readings, practical exercises, and computer simulations. Two studies did not offer any further information regarding the type and structure of the tasks. Bux and van Vuuren (2019) only pointed out that they analyzed two interventions (one longer and one shorter) that were part of the Junior Achievement South Africa (JASA) program. Volery and colleagues (2013) examined three different entrepreneurship programs which had similarities in terms of type and structure of the tasks. Overall, the sample covered three different entrepreneurship programs: (1) an intervention that was offered by the Swiss Federal Office for Professional Education and Technology, (2) the ‘Company Program’ of Youth Enterprise Switzerland (a member of Junior Achievement Worldwide), and (3) the ‘Learn to undertake’ program. All of these programs included, for example, field trips, development of business ideas, and consultation with practitioners, all focused on ‘venture creation’. They were carried out as a program separate from the actual teaching. The transnational European entrepreneurship program ‘Youth Start—Entrepreneurial Challenges’ (Streicher et al. 2019), however, has the ability to embed EE in school curricula through structured teaching units suitable for different topics and in alignment with standard learning goals. Therefore, the learning units consist of tasks with different content focus, different degrees of complexity, and different requirements in terms of amount of time. Overall, while none of the studies explicitly focused on the analysis of specific learning strategies, learning arrangements, or designs of the teaching context, implicitly, there was a tendency towards the use of student-oriented (Weimer 2002), active learning approaches in the analyzed interventions.

Fourth, regarding *specific outcomes* that the focal program sought to promote, all programs included a focus on the development of multiple distinct outcomes. According to Longva and Foss (2018), these can be characterized as cognitive (e.g., knowledge), skill-based (e.g., business modeling, teamwork), affective (e.g., passion), conative (e.g., ESE, entrepreneurial intent), or behavioral (e.g., employability). Most interventions aimed at several outcomes at the same time, including cognitive outcomes like entrepreneurial knowledge such as business plans or basics of accounting (Sánchez 2013), skill-based outcomes like teamwork (e.g., Rosendahl Huber et al. 2014), or affective outcomes such as risk-taking (e.g., Cárcamo-Solís et al. 2017; Sánchez 2013; Volery et al. 2013) and, of course, conative and attitudinal ones such as ESE. Thus, the interventions tended not to aim at the promotion of ESE alone, but more comprehensively of a whole range of outcome variables that are desirable from an EE viewpoint.

#### Results of comparing the studies’ findings

Finally, we considered the studies’ *findings regarding the effectiveness of the interventions in promoting the conative outcome ESE* (cf. Longva and Foss 2018): Six studies reported a generally positive influence of the interventions (Bux and van Vuuren 2019; Cárcamo-Solís et al. 2017; Grewe and Brahm 2020; Rosendahl Huber et al. 2014; Sánchez 2013; Streicher et al. 2019). According to Bux and van Vuuren (2019), an analogous longer-term intervention had a stronger positive effect than a shorter one on ESE. Yet, the lack of a control group, a pretest, and detailed information regarding the differences between the interventions, imply that the implications of this result are unclear. Bergman et al. (2011) did not find a significant overall rise in ESE but observed a significant effect conditional on gender: Before the intervention, girls had a higher level of ESE than boys. Afterwards, ESE had increased significantly for boys. For girls, ESE actually declined when comparing pre- and post-intervention scores. The results have to be interpreted with some caution, though, due to a substantial decline in the number of participants from measurement at time 1 (*n* = 881) to measurement at time 2 (*n* = 266). Finally, Volery and colleagues (2013) did not find a significant (within-subject) change in ESE at all (Volery et al. 2013). In a similar vein, Grewe and Brahm (2020) did not find a significant difference between treatment and control group (i.e., a regular economics class). In addition, beyond focusing on the main effect, some studies reported further moderating influences. Streicher and colleagues (2019) found, next to a positive overall effect, that participants who had prior experiences in entrepreneurial activities had the highest increase in ESE.

Also, we considered effect sizes for those studies that reported a significant main effect of the focal intervention(s) on ESE. Four of these studies did not provide any such information (Bux and van Vuuren 2019; Cárcamo-Solís et al. 2017; Grewe and Brahm 2020; Sánchez 2013). In the other two studies, treatment effects were substantial and of a similar magnitude: Rosendahl Huber and colleagues (2014) found children participating in the intervention to show a significant increase in ESE (short-term assessment) of 0.16 of a standard deviation compared to the control group. The effect size was similar in magnitude to Streicher et al. (2019), who reported a value of 0.177. Table 2 offers an overview of the interventions’ content and duration.

**Table 2:** Overview of the interventions’ content and duration in EE research

Author(s) (year)	Impact	Duration	Content: Analysis of (the)…
Bergman et al. (2011)	No overall effect but gender moderation (positive effect for male participants)	12 months	… program 'Young Entrepreneurs—Israel' in which the students set up their mini-company and acquired entrepreneurial skills (e.g., managing market research or product development). At the end of the year, they competed with other students in a regional, national, or international competition
Bux and van Vuuren (2019)	Positive, and more so for students in the long-term intervention	n.a	… two Junior Achievement South Africa (JASA) entrepreneurship education interventions (no further information provided)
Cárcamo-Solís et al. (2017)	Positive	n.a	… Mexican program ‘My first Company: Entrepreneurship by playing for primary school children’ in which the participants founded a fictitious mini-company, took over various business positions, and presented their entrepreneurial ideas to stakeholders
Grewe and Brahm (2020)	Positive, but no difference from control group (regular economics class)	9 months	… JUNIOR mini-company program in which the students develop an idea, found and organize an own company, raise seed capital, execute and administrate the production, marketing and sales and finally close their company
Rosendahl Huber et al. (2014)	Positive	2–4 weeks	… ‘BizWorld’ Program in which the students worked in groups of five to six people, assigned a business role, founded a fictitious company, developed a product and a marketing campaign, formulated a business plan, and presented their goods to potential customers. In addition, the teams competed with each other and were rewarded with an official certificate
Sánchez (2013)	Positive	8 months	… Spanish program ‘E-Vitamin’ which has not only a focus on 'acting like an entrepreneur' (e.g., writing a business plan) but also on generating a link to practice, which was, e.g., implemented through discussions with entrepreneurs
Streicher et al. (2019)	Positive	12 months	… transnational European entrepreneurship program ‘Youth Start—Entrepreneurial Challenges’ in which the participants completed challenges along with the three themes entrepreneurial core skills, culture, and civic education
Volery et al. (2013)	No effect	6–12 months	… three Swiss programs for the development of entrepreneurial knowledge and skills. The authors do not provide any additional information about the structure of the individual programs. In addition, the intervention content differs fundamentally, e.g., in terms of the overall duration, the implementation by various instructors, or the voluntary versus mandatory nature of the interventions

The table provides an overview of the interventions in EE research with a focus on the effect on the promotion of ESE, the duration, and the content of the interventions

### Summary of the comparison: tentative implications and open questions

Overall, extant school interventions during primary and secondary education mostly appear to be effective in promoting ESE. Given the substantial variety of these interventions, this result seems fairly robust across different types of programs. In further support of this robustness, most studies used standard experimental or quasi-experimental designs. With one exception (Bux and van Vuuren 2019), they all used a pre-posttest design. Volery and colleagues (2013) even examined long-term effects (pre-, post-, post-posttest design). All studies except for two (Bux and van Vuuren 2019; Cárcamo-Solís et al. 2017) used at least one treatment and one control group.

Beyond this encouraging basic result, the review also revealed several important but as yet unaddressed gaps in the literature. First, despite the broad robustness of the results, the evidence is not entirely conclusive: One study (Volery et al. 2013) failed to find a significantly positive effect of the focal interventions, without any apparent reasons. Another study (Bergman et al. 2011) found a gender-dependent effect of the focal intervention: the positive effect of the intervention on ESE was restricted to boys. Bergman and colleagues (2011) tentatively attributed the observed decline in girls’ ESE and the simultaneous increase in boys’ ESE to the competitive character of the focal intervention. Games preferred and played by girls tend to be significantly less competitive than those preferred and played by boys (e.g., Weinberger and Stein 2008), and boys tend to have a higher willingness to compete. The competitive nature of the program assessed by Bergman and colleagues (2011), thus, likely appealed more to boys than to girls (cf. Gneezy et al. 2003). Future research into gender-specific effects depending on the design of interventions, hence, seems important in order to reach both genders through early EE interventions. In addition, other moderating variables might be relevant, too, such as socio-economic background or prior entrepreneurial experience (e.g., Streicher et al. 2019).

Second, even if various types of intervention all ‘work’—which works best, and at what cost? Considering costs: All of the programs were (as far as information was provided) fairly long-term oriented. To what extent could ESE be positively affected also by more short-term oriented interventions? Short-term oriented interventions might be easier to integrate into the compulsory parts of the curriculum, ultimately providing more universal access across types of schools and individuals. They are also likely to exert less of a strain on scarce public resources and limited teaching time. Considering effectiveness: All of the studies tended towards considering the effectiveness of the intervention as a whole in terms of the ‘input’ it provided, including also other outcome variables such as entrepreneurial intentions or knowledge of entrepreneurial processes. There are good reasons for adopting such a comprehensive approach. However, it makes it difficult to pinpoint the precise mechanisms that bring about the envisaged increase in ESE, and, thus, hinders the development of more targeted interventions.

Third, this research gap is closely related to a common feature across all eight studies: a focus on assessing the effects of interventions established earlier and for purposes other than the focal studies themselves. An integrative approach, offering both development of an intervention and a systematic assessment of its effectiveness is apparently uncommon in EE research, to date.

Fourth, while the reviewed studies were implemented in a variety of countries, the question whether country-level factors (e.g., culture, broader institutional or economic context) might impact the effectiveness of whole interventions or specific elements has remained virtually unaddressed, to date. Moreover, most studies were conducted with an emphasis on European countries. Only Cárcamo-Solís and colleagues (2017) and Bux and van Vuuren (2019) analyzed intervention effectiveness in emerging market contexts (Mexico and South Africa, respectively). Given that, for example, the ‘BizWorld’ program (see Rosendahl Huber et al. 2014) has meanwhile been implemented in more than 100 countries, covering more than 68,000 students according to the program’s website (https://bizworld.org/; as of October 19, 2021), including implementations in, for example, Nigeria and Egypt, broadening the scope of the investigation to include other countries/regions seems feasible.

Overall, thus there seems to be a need for future comparative research (e.g., regarding the effectiveness of specific interventional elements such as duration or gender-specific designs). In order to inspire future research in this respect, we propose to engage in an entrepreneurial activity, that is: ‘thinking out of the box’. Specifically, we suggest ‘thinking in other boxes’, by drawing on research on *intervention-based promotion of STEM-related self-efficacies*.

## The promotion of STEM-related self-efficacy in primary and secondary education

### Methods

To identify relevant studies from the field of STEM education research, we used a similar approach as in the first part (Fisch and Block 2018; Short 2009). Next to databases like Web of Science or Google Scholar, we used STEM-specific databases like MathSciNet. As we were interested in interventions aiming at STEM-related self-efficacies, we expanded our search accordingly. We integrated the keywords ‘science’, ‘technical’, ‘engineering’, ‘mathematics’, ‘computer’ or ‘STEM’ in different combinations with ‘self-efficacy’ and terms like ‘school’, ‘pupils’, ‘students’, ‘education’, ‘experiment’ or ‘quasi-experiment’. In addition, the inclusion criteria were applied analogously to the first part of the study. Overall, we found 561 papers. After the exclusion process, we were left with 17 studies.

### Literature review: STEM education research

**Fig. 2:**
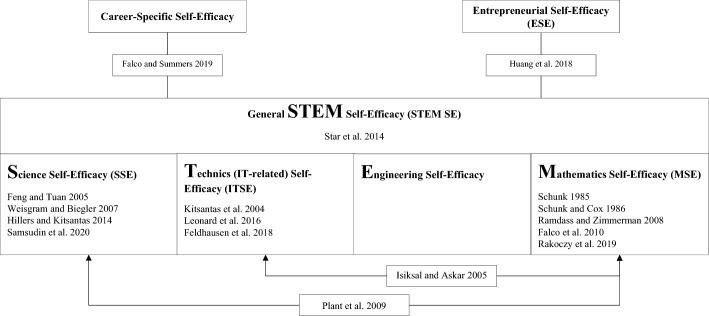
Overview of the interventions belonging to STEM education research

Interventions in the reviewed studies were concerned with the promotion of different STEM-related self-efficacies: Five studies addressed mathematics self-efficacy (MSE) (Falco et al. 2010; Rakoczy et al. 2019; Ramdass and Zimmerman 2008; Schunk 1985; Schunk and Cox 1986), four addressed science self-efficacy (SSE) (Feng and Tuan 2005; Hiller and Kitsantas 2014; Samsudin et al. 2020; Weisgram and Bigler 2007), three analyzed IT-related self-efficacy (ITSE) (Feldhausen et al. 2018; Kitsantas et al. 2004; Leonard et al. 2016). Two further studies focused on multiple STEM-specific self-efficacies simultaneously (STEM SE): MSE and ITSE (Isiksal and Askar 2005), and MSE and SSE (Plant et al. 2009). One study considered a generalized STEM self-efficacy (STEM SE) (Star et al. 2014). Finally, two studies employed an interdisciplinary approach: Falco and Summers (2017) focused on the promotion of MSE and SSE and, in addition, on career-specific self-efficacy. Huang et al. (2018) considered the intersection of STEM and entrepreneurial research and analyzed STEM SE and ESE in the context of a STEM career-based intervention. Figure 2 provides an overview.

We compared the studies with respect to (1) research design, (2) theoretical frameworks, (3) structure and content of the interventions, and (4) the findings—with an emphasis on the issues pointed out as open questions emanating from the review of the EE literature.

#### Results of comparing research designs

Again, we compared the studies with respect to (i) whether or not development of the focal intervention had been an *integral part of the study*, (ii) the *number of interventions* covered by study, and (iii) the use of either an *experimental or a quasi-experimental design*, and (iv) whether or not they offered a *comparison across multiple, different interventions*.

First, unlike the EE studies, all 17 STEM studies analyzed the effects of interventions that were an *integral part of the studies* themselves. That is, they were designed specifically for the purpose of the corresponding studies in order to promote the outcomes assessed in the studies.

Second, in terms of *number of interventions*, the majority of studies (*n* = 13) investigated the influence of a single program. Three studies focused on two different programs (Feldhausen et al. 2018: Scratch; Isiksal and Askar 2005: spreadsheet and geometry software; Leonard et al. 2016: game design and robotics). Star and colleagues (2014) analyzed three different game-based learning programs. Since the promotion of STEM SE was only the focus of the sub-program ‘Immersive Virtual Environment’ (IVE), only this part of the study is relevant and covered here. Also, as in EE research, all programs were offered at the school level on a voluntary basis. Six studies (Falco and Summers 2017; Feldhausen et al. 2018; Hiller and Kitsantas 2014; Huang et al. 2018; Leonard et al. 2016; Weisgram and Bigler 2007) analyzed interventions that were voluntary also for participants at the individual level, while 11 studies analyzed interventions that were compulsory for students, provided their school had decided to take part in the program.

Regarding the use of *experimental* or *quasi-experimental designs*, nine studies used experimental designs, while seven of them analyzed quasi-experiments. One study (Rakozcy et al. 2019) used a randomized field trial. 14 studies used a pre-posttest design. Two studies used solely a post-questionnaire (Huang et al. 2018; Plant et al. 2009), implying that it is difficult to establish whether the observed effects were triggered by the interventions. Like in EE research, only one study (Falco et al. 2010) made use of a pre-, post-, and post-posttest design. Regarding the use of control groups, we identified different approaches. Eight studies used a classic design (treatment and control group). One study (Huang et al. 2018) did not include a control group. Thus, identification of causal mechanisms is, strictly speaking, not possible. Yet, the STEM studies also included a further type of design, beyond the designs employed in EE research so far: Eight studies (Feldhausen et al. 2018; Isiksal and Askar 2005; Kitsantas et al. 2004; Leonard et al. 2016; Plant et al. 2009; Schunk 1985; Schunk and Cox 1986; Star et al. 2014) simultaneously analyzed several treatment groups, ranging from two (Feldhausen et al. 2018) to nine treatment groups (Schunk and Cox 1986). So, in STEM education research it appears to be more common to employ designs that integrate multiple different treatments within a focal intervention.

Moreover, we compared the studies based on whether or not they offered a *comparison across multiple, different interventions*. With the exception of Star and colleagues (2014), Isiksal and Askar (2005), Feldhausen and colleagues (2018), and Leonard and colleagues (2016), who analyzed different interventions in a single country context, all studies analyzed a single intervention (some of them including several treatments, though) in a single country context. Ten studies were conducted in the U.S. (Falco and Summers 2017; Feldhausen et al. 2018; Huang et al. 2018; Kitsantas et al. 2014; Leonard et al. 2016; Plant et al. 2009; Ramdass and Zimerman 2008; Schunk 1985; Schunk and Cox 1986; Star et al. 2014) two studies in Asia (Taiwan: Feng and Tuan 2005; Malaysia: Samsudin et al. 2020), one study in Germany (Rakoczy et al. 2019), and one study in Turkey (Isiksal and Askar 2005). For three studies (Falco et al. 2010; Kitsantas et al. 2004; Weisgram and Bigler 2007), information regarding the country setting is lacking. Table 1 contains as well an overview of the research designs used in STEM education research.

#### Results of comparing the theoretical frameworks

Related to the theoretical framework, studies varied considerably. Six studies (Feldhausen et al. 2018; Feng and Tuan 2005; Huang et al. 2018; Kitsantas et al. 2004; Leonard et al. 2016; Weisgram and Bigler 2007) completely dispensed with any direct reference to the theoretical construct SE; six studies (Falco et al. 2010; Isiksal and Askar 2005; Rakoczy et al. 2019; Ramdass and Zimmerman 2008; Schunk 1985; Schunk and Cox 1986) discussed the state of research regarding the construct itself and explained the term SE. The remaining five studies used SE-based theories as their theoretical framework. Star and colleagues (2014) and Samsudin and colleagues (2020) referred to Bandura’s social learning theory (1977, 1986) and considered the four different *sources of SE* in detail: ‘mastery experiences’ as the interpreted results of past performances; ‘vicarious experiences’ through observation (and imitation) of (successful) others in similar situations; ‘verbal persuasion’ (talks with others about own abilities); ‘physiological and affective states’ (e.g., anxiety or stress) (e.g., Bandura and Schunk 1981). Plant and colleagues (2009) drew on Bandura’s social learning theory, focusing explicitly on ‘vicarious experiences’. As in EE research, two studies (Falco and Summers 2017; Hiller and Kitsantas 2014) focused on career choice decisions and referred to the SCCT (Lent et al. 1994).

#### Results of comparing the structure and content of the interventions

Guided by the research gaps identified based on the review of EE studies, we examined in particular (i) the overall *duration of the interventions* and *the number and duration of the individual sessions*, (ii) the *design of the teaching context* and the use of *specific learning strategies*, and (iii) *gender-specific intervention designs*.

First, the programs differed in terms of *overall duration* and *number and duration of the individual sessions*. Across all studies, a temporal span of one day (Kitsantas et al. 2004; Plant et al. 2009; Weisgram and Bigler 2007) up to 3 years (Huang et al. 2018) was observed. Five interventions were designed for a duration of fewer than ten days. Six programs lasted more than ten days. Samsudin and colleagues (2020) did not reveal detailed information about the overall duration or number and duration of individual sessions. Due to the content (designing and building a pulley system) and the inclusion of many subtasks, it seems likely, however, that this intervention would have consisted of a teaching unit with several sub-sessions.

Concerning the duration of individual sessions, ten studies provided detailed information. With a duration between 30 and 60 min (seven studies), the basis of these individual sessions was typically a standard school lesson (excluding the studies by Feldhausen et al. 2018; Leonard et al. 2016; Huang et al. 2018). The interventions overall covered between one and several weeks and included usually between four and 13 individual sessions (Falco et al. 2010; Falco and Summers 2017; Feldhausen et al. 2018; Feng and Tuan 2005; Huang et al. 2018; Isiksal and Askar 2005; Kitsantas et al. 2004; Leonard et al. 2016; Schunk 1985; Schunk and Cox 1986; Star et al. 2014). Star and colleagues (2014) did not provide any information regarding the duration of individual sessions but described the program as a ‘four-day program with four sessions’.

Second, we compared the studies based on the *design of the teaching context* and the use of *specific learning strategies*. Next to five studies (Falco and Summers 2017; Feldhausen et al. 2018; Huang et al. 2018; Plant et al. 2009; Weisgram and Bigler 2007), which focused on the effectiveness of a comprehensive program—similar to the entrepreneurial interventions—, four studies (Feng and Tuan 2005; Hiller and Kitsantas 2014; Leonard et al 2016; Samsudin et al. 2020) zoomed in on details of the *teaching context*, that is, on specific pedagogical approaches or psychological models adopted by the teachers in order to structure the intervention. Feng and Tuan (2005) analyzed as focal aspect of the teaching process the effects of a *motivational-based learning environment.* They designed a teaching unit (on bases and acids [chemistry]), referring to the psychology-based ARCS model of instructional design (Keller 1987). Following the ARCS model, in order to positively affect students’ motivation, content and setting should be designed so as (1) to attract the interest of the students (Attention), (2) to be relevant for them (e.g., passing exams or importance for their own lives) (Relevance), (3) to facilitate the development of positive expectations regarding learning outcomes (Confidence), and (4) to be supported by intrinsic and extrinsic reinforcers, such as positive feedback or material recognition (Satisfaction). Hiller and Kitsantas (2014) also considered motivational factors and investigated, specifically, the effects of a ‘Citizen Science Program’. Generally, such science-oriented programs are employed in school contexts with the aim to increase students’ interest in STEM-related careers: Volunteer students collect data for professional scientists, mostly with a focus on animal populations or environmental factors. From a pedagogical viewpoint, this type of program seeks to foster project- and problem-based learning and the establishment of linkages between the subject matter and students’ daily lives. Hiller and Kitsantas (2014) examined such a program related to the horseshoe crabs. Students worked together with field experts, were involved in active field research, and collected data on horseshoe crabs for a professional biologist. Samsudin and colleagues (2020) similarly examined a science-oriented program with a focus on project-based learning. According to this approach, students work together in groups and solve authentic real-world problems (Blumenfeld et al. 1991). The students experienced the mechanical structure of a pulley system by exploring and building the fundamental functionality of a crane. Leonard and colleagues (2016) dealt with the question of designing a learning environment. They used the technology design framework ‘learning-for-use’ approach to implement interventions with a focus on robotics applications and game design, an approach which focuses on cognitive processes of learning. In so doing, they emphasized the incrementality of knowledge construction, i.e. the need for newly-to-be-acquired knowledge to be linked to prior knowledge.

Six studies (Falco et al. 2010; Kitsantas et al. 2004; Schunk and Cox 1986; Rakoczy et al. 2019; Ramdass and Zimmerman 2008; Schunk 1985) focused in-depth on the effectiveness of *specific learning strategies* that can be incorporated (and potentially combined) within teaching contexts in order to support students’ effective learning. In so doing, they tended to make use of multiple treatment groups. Four studies investigated different approaches of ‘self-regulation’ as specific learning strategy. Self-regulation of learning processes supposedly leads to a more accurate perception and assessment of one’s own abilities and a superior reflection of the learning progress. In the study of Ramdass and Zimmerman (2008), students learned such a strategy for self-regulation (self-correcting mathematical answers) and had a checklist for self-correcting their own solutions*.* Rakoczy and colleagues (2019) focused on the effectiveness of formative assessments. In a teaching unit on the ‘Theorem of Pythagoras’, students received process-oriented feedback, which combines feedback at the task level (information on task performance), process level (information on the process to master a task), and self-regulatory level (information on the regulation of action) (Hattie and Timperley 2007). Process-oriented feedback should, on the one hand, help students by providing knowledge about weaknesses and knowledge about strategies to solve tasks. On the other hand, it fulfills motivational functions, like enhancing self-efficacy by making it possible to master tasks (Narciss 2008). Next to a focus on effort-attributional feedback, Schunk and Cox (1986) focused on the learning strategy ‘overt verbalization’, which has also a self-regulation function. Students should benefit from explicit strategy training in verbalizing aloud each solution step. ‘Overt verbalization’ arguably can help students to concentrate on important issues of the tasks at hand and, furthermore, offers students greater control over their own learning process. Kitsantas and colleagues (2004) analyzed the effectiveness of self-evaluation strategies, in conjunction with various ‘written signals’ such as, for example, headings in instructions or short summaries at the end of a text, as well as the learning strategy ‘goal setting’ (e.g., Ames and Archer 1988). According to this learning strategy, goals can be either ‘process-’ or ‘outcome-’ oriented, with process goals being expected to lead to a superior learning process and, ultimately, better performance. Two more studies concentrated on the use of the learning strategy ‘goal setting’. Falco and colleagues (2010) analyzed the effectiveness of ‘goal setting’ and ‘planning’ as one of several treatments, in which students were trained in relation to ‘time-management’, ‘goal setting’, specific mathematic-related learning skills (‘mathematics study skills’), and ‘help-seeking’. Schunk (1985) also considered ‘goal setting’ as learning strategy, focusing on the temporal distance of reaching a goal, with ‘proximal goals’ being expected to result in higher motivation than goals in a distant future (Bandura and Schunk 1981; Manderlink and Harackiewicz 1984). Specifically, the study compared the effectiveness of proximal goals set by students themselves compared to those set by teachers. Table 3 provides an overview of the interventions including their overall duration and content.

**Table 3:** Overview of the interventions’ content and duration in STEM education research

Author(s) (year)	Impact	Duration	Content: analysis of …
Falco et al. (2010)	Positive, especially for women	9 weeks	… different learning strategies like ‘goal setting’, ‘planning’, ‘time-management’, and ‘help-seeking’ during math learning
Falco and Summers (2017)	Positive for STEM SE and career-specific SE	9 weeks	… an occupational program (focus on STEM), including the situation of the labor market, working conditions, gender-specific barriers
Feldhausen et al. (2018)	Positive for computer programming SE, no influence on problem-solving SE	4 weeks	… of the two programs ‘Saving the Martian’ (5th/6th grade) and ‘Mighty Micro Controllers’ (7th to 9th grade), both with a focus on programming
Feng and Tuan (2005)	Positive	multiple weeks	… a unit on acids and bases based on the ‘ARCS model’ by Keller (1987) (Attention, Relevance, Confidence, Satisfaction)
Hiller and Kitsantas (2014)	Positive	1 day	… practically-oriented ‘Citizen Science Program’ (focus on horse-shoe crabs), a program in which students worked together with biologists, collected bio-data, were involved in active field research
Huang et al. (2018)	Positive	3 years	… ‘STEM-Inc.’, a program which offers insights into the STEM and entrepreneurial career field
Isiksal and Askar (2005)	Positive for MSE (especially in dynamic geometry software intervention), gender differences in ITSE (higher for men)	3 weeks	… different programs for the promotion of mathematical skills with a focus on the use of a spreadsheet program and a dynamic geometry software
Kitsantas et al. (2004)	Positive, especially for groups with a focus on ‘process goals’ and ‘self-evaluation strategies’	3 days	… the effectiveness of the specific learning strategy ‘goal setting’ as well as two influencing factors 'self-evaluation strategies' and 'written signals' (e.g., headings or summaries) during a computer-based program for the designing of a computer presentation
Leonard et al. (2016)	Positive, especially for robotics/game design intervention	6–10 weeks	… interventions with a focus on robotics, game design, and a combination of both
Plant et al. (2009)	Positive, especially for interaction with a female interface agent	1 day	… the interaction with an animated interface agent (male or female) as social models for changing students’ attitudes towards the engineering-related field
Rakoczy et al. (2019)	Positive for direct effect of formative assessment on SE, no evidence for mediation effect of ‘perceived usefulness’ on SE	3 weeks	… a teaching unit on the ‘Theorem of Pythagoras’ with a focus on the effectiveness of process-oriented feedback
Ramdass and Zimmerman (2008)	Positive	4 days	… the specific learning strategy of ‘self-regulation’ during the division learning process
Samsudin et al. (2020)	Positive	n.a	… project-based teaching unit (focus on designing a pulley system)
Schunk (1985)	Positive for students who set their own goals	1 week	… effect of ‘goal setting’ (focus on ‘proximal goals’ [own goals versus goals set by teachers versus no goals) as a specific learning strategy during a subtraction-training program
Schunk and Cox (1986)	Positive	6 days	… the specific learning strategies ‘overt verbalization’ and effort-attributional feedback during the learning of algorithmic knowledge
Star et al. (2014)	No Impact	4 days	… the game-based learning program ‘Immersive Virtual Environment (IVE), where the participants solve mathematical problems for the promotion of mathematical motivation
Weisgram and Bigler (2007)	Positive	1 day	… one-day-program to increase girls' interest in science, including a lesson about gender-based occupational discrimination (comparison with program without gender-based occupational discrimination)

The table provides an overview of the interventions in STEM education research with a focus on the effect on the promotion of STEM SE, the duration, and the content of the interventions

Third, we consider whether the studies employed *gender-specific intervention designs.* Two studies developed and analyzed interventions that were geared explicitly at promoting the participation of women in STEM professions. Weisgram and Bigler (2007) focused on the consequences of learning about gender-based occupational discrimination on various outcomes. In addition to a one-day program to promote girls’ interest in the sciences, the participating girls received an additional intervention on the topic of gender discrimination, aimed at promoting girls’ interest in, and positive attitudes towards science. Falco and Summers (2017) used a cross-disciplinary approach at the interface of STEM and career choice research. Girls took part in lessons that specifically addressed the issue of STEM careers for women, including labor market conditions, working conditions, and gender-specific barriers.

#### Results of comparing the studies’ findings

In terms of *findings*, in general, first, most of the studies succeeded in promoting the focal STEM-related self-efficacies. From among the studies that assessed the effectiveness of either a comprehensive program or particular teaching context, only the ‘Immersive Virtual Environment’ program (Star et al. 2014), where the students explored a virtual reality and solved mathematical problems, appeared ineffective. In discussing possible reasons, Star and colleagues (2014) focused on the short duration (4 days) in relation to the particularly high complexity of the intervention in terms of cognitive and temporal resources. As a comparison with the other 16 studies covered in this review shows that other interventions designed for similarly short durations (e.g., Hillers and Kitsantas 2014; Kitsantas et al. 2004; Schunk 1985; Weisgram and Bigler 2007) were, in contrast, able to promote focal STEM-related self-efficacies despite similarly short durations, the complexity of the application indeed appears to have been a major obstacle to the intervention’s effectiveness.

As to be expected, given the number of treatments, the results are somewhat more complex for the studies that assessed the effectiveness of specific learning strategies. Three studies found the learning strategy ‘self-regulation’ to be generally effective at raising focal STEM SEs (Kitsantas et al. 2004; Ramdass and Zimmerman 2008; Schunk and Cox 1986). Also, in support of the effectiveness of the focal learning strategy, Schunk (1985) found that students who set their own goals (treatment: proximal goals, set by students themselves) had the highest increase in MSE, while there was no significant difference between the control group (no goals group) and the second treatment group (proximal goals, set by teacher). Also, in line with expectations, Kitsantas and colleagues (2004) found the use of process goals to lead to a significantly higher increase in ITSE than the use of outcome goals. Also, effort-attributional (Schunk and Cox 1986) and process-oriented feedback (Rakoczy et al. 2019) were effective.

Finally, interesting results emerged in relation to the moderating effect of gender. Isiksal and Askar (2005) who analyzed different software programs showed—next to a generally positive influence of the interventions on ITSE—a higher increase in ITSE for male than for female participants in response to the focal intervention. They explained this effect as a result of boys’ generally higher interest in computer tasks. Interestingly, they did not observe a significant difference in MSE gain across boys and girls in response to the intervention. Plant and colleagues (2009) analyzed the effect on students’ attitudes towards engineering-related fields of using animated (male and female) interface agents as social models: Compared to the no-agent control condition, both the female and male interface agents raised participants’ engineering-related self-efficacies (MSE and SSE)—for both female and male participants—, with the female agent being somewhat more effective, but not significantly so. Falco and colleagues (2010) found that girls had significantly lower MSE than boys prior to the intervention but in the experimental group, they experienced the highest increase in MSE among all participants, confirming the effectiveness of the intervention especially for this group. Further, Falco and colleagues (2010) found a difference in students’ MSE, depending on attributes of their class teachers.

### Summary of the comparison

The analyzed interventions appear to have been generally effective in promoting STEM-related self-efficacies, and fairly robustly so across different programs, intervention durations, teaching contexts, and learnings strategies. Yet, some of them seem to have been more effective than others, especially for certain target groups (e.g., girls versus boys), suggesting that a nuanced assessment may be most appropriate. Design-wise, major similarities across the reviewed studies further concerned (a) a simultaneous focus on developing and on assessing interventions, (b) an inclusion of multiple treatment groups, and (c) a lack of larger numbers of observations across a variety of contexts.

## Implications for future research on early EE

Jointly considering the reviewed studies in EE and STEM education enables us to derive implications for future EE research by referring both to the research gaps identified based on the comparison of EE studies, and by proposing ways in which STEM research may inspire future studies on early EE. The most basic suggestion from the consideration of both literatures is, that future EE research may benefit, as a complementary approach, from the joint inclusion of development and assessment of interventions, an approach that is common in STEM research and virtually absent from EE research to date. Such an integrative approach would allow scholars to address under-explored issues, such as (i) possible moderating effects (e.g., gender, social background), (ii) the comparative effectiveness of short-term interventions that could be implemented fairly easily into compulsory school curricula, (iii) the influence of specific learning strategies, teaching models, and media, and (iv) the use of different experimental and control groups.

### Moderating effects

In terms of a possible moderation effect of gender, in the EE literature, Bergman and colleagues (2011) observed that the effectiveness of the focal intervention in encouraging ESE strongly depends on the gender of participants, with girls’ ESE actually decreasing in response. Bergman and colleagues (2011) tentatively attributed the lack of a gender-independent positive effect of the intervention on ESE in their study to the highly competitive nature of their intervention. Indeed, the focal interventions assessed in the other studies either did not explicitly include competitive elements at all or did so to a much lesser degree (Rosendahl Huber et al. 2014). Thus, the extent to which an early entrepreneurship program emphasizes competition as a core element could possibly represent a major determinant of whether a gender moderation is to be expected. Given the importance of this issue, especially in terms of employing interventions that realistically prepare students for their future professional lives, it seems imperative that future research on early EE might seek to more comprehensively and systematically address this issue. Integrating the design of a focal intervention and its assessment within a single study, as it is common in STEM research, would greatly facilitate this analysis. In addition, STEM research suggests that other features of the interventions, beyond the degree of competitiveness, may also affect the gender-specific effectiveness of a program. Isiksal and Askar (2005), for example, found that the use of different media (e.g., geometry software, spreadsheet program) might have a distinct influence on the effectiveness of interventions, with boys, in particular, tending to benefit from computer-based vis-à-vis traditional interventional designs. Overall, STEM education research thus corroborates the importance of gender and associated role models and socialization patterns, and, further, suggests that future EE research may benefit from more comprehensively investigating the influence of these factors in relation to both the intervention as whole and to specific characteristics of the intervention (e.g., degree of competitive orientation, thematic context such as STEM and, within STEM, various media). Indeed, a recent review of the impact of role models in entrepreneurship (Abbasianchavari and Moritz 2021) underlines both the importance of role models as such for various entrepreneurial outcome variables, as well as the relevance of gender for the effects of role models. Additionally, it suggests that gender-dependent effects of role models may further interact with other characteristics such as success or failure, or domain of an entrepreneurial endeavor, or cultural background of the students who experience an intervention. In exploring possible gender moderations, it might well turn out that also other individual-level factors (e.g., social background, prior learning experiences) might exist that could similarly exert moderating effects, but have remained unaddressed so far. Further, the STEM studies additionally suggest that not just the gender of participants (Falco et al. 2010; Plant et al. 2009; Isiksal and Askar 2005) might influence an intervention's effectiveness, but also potentially the specific attributes (including gender) of the person administering the intervention (e.g., Falco et al. 2010)—a type of (gender) moderation that EE research still has to account for comprehensively and systematically. A more encompassing analysis of moderating effects is not only valuable from an academic viewpoint (see e.g., for the relevance of gender differences in EE interventions at the university level: Padilla-Angulo et al. [in press]). It is also a socio-political imperative: Interventions could be tailor-made to target specific groups (e.g., girls, students with distinct thematic orientations (e.g., STEM, arts), socially disadvantaged children).

### Comparative effectiveness of short-term versus longer-term interventions

**Fig. 3:**
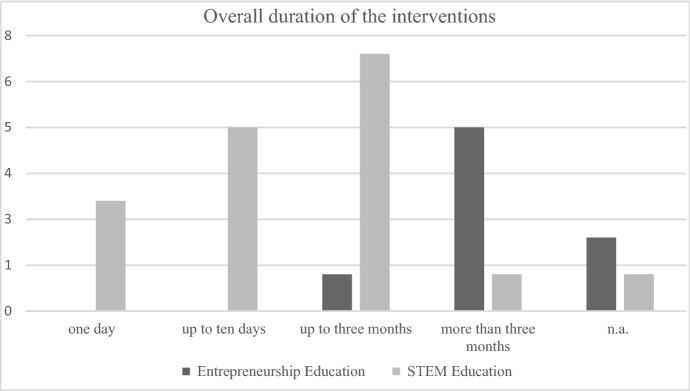
Duration of the programs, entrepreneurship versus STEM education research

In the EE literature, Bux and van Vuuren (2019) compared a short-term and a long-term version of an intervention. While the long-term version seemed more effective at promoting ESE, the short-term version raised ESE, too. The question, thus, is whether short-term interventions can be sufficient. While the focal interventions evaluated in the EE literature were mostly designed for longer time periods, many interventions investigated in STEM education research were more short-term oriented, some lasting only for a day (e.g., Hiller and Kitsantas 2014; Plant et al. 2009; Weisgram and Biegler 2007) or a few school lessons scattered across several weeks (e.g., Falco et al. 2010; Ramdass and Zimmerman 2008; Schunk 1985) (see Fig. 3 for an overview).

Yet, nearly all of these short-term interventions appear to have been successful in promoting STEM-related self-efficacies, despite their limited duration. This result tentatively indicates that even short-term interventions provided they are properly designed, may be able to foster the development of specific self-efficacies. Assuming that short-term interventions are much easier to integrate into the compulsory parts of school curricula and provide more universal access across types of schools and individuals, future interventions in EE may find it worthwhile to investigate the effects of shorter interventions in general, and, in particular, concerning ESE—even if that requires developing interventions specifically for the purpose of the focal study.

### Specific learning strategies, teaching models, and media

Complementing the typical approach in EE research of evaluating the outcomes of established, comprehensive interventions ‘as a whole’, our review of STEM research suggests to complement this focus by investigating also the influence of individual components of such overarching interventions, that is, specific learning strategies, of various teaching contexts and models (e.g., traditional teaching versus teamwork), and the use of different media (similarly, Padilla-Angulo and colleagues (in press) advocate disentangling the effectiveness of individual academic activities of EE the university level). The reviewed STEM studies provide valuable insights into the most promising individual educational elements for future EE research to consider, as most of them zoomed in on fine-grained pedagogical aspects. While most studies found general support for the effectiveness of the focal interventions, comparisons across different specific learning strategies, for example, showed that some of them were significantly more effective than others. In particular, the use of self-regulation strategies (e.g., Ramdass and Zimmerman 2008) or different types of feedback (e.g., Rakoczy et al. 2019; Schunk and Cox 1986) appeared to be highly effective. Also, several STEM studies explicitly incorporated different treatments based on various media in their focal interventions (Feldhausen et al. 2018; Isiksal and Askar 2005; Plant et al. 2009; Star et al. 2014) and found differences in terms of effectiveness. Isiksal and Askar (2005), for example, observed that, unlike a spreadsheet-based instruction, use of a dedicated geometry software was relatively more effective in stimulating the development of MSE. In sum, future studies in EE should find it worthwhile to systematically vary learning strategies, teaching models, and media in order to uncover what works best, and for which target groups, for stimulating ESE in primary and secondary education.

### Experimental and control groups

Regarding experimental conditions, most EE studies have, to date, adopted designs with one treatment and a control group. In STEM research variation of multiple pedagogical features within an intervention by means of several treatments is common. EE studies may find this approach similarly valuable, especially when seeking to disentangle more and less effective ways (e.g., in terms of learning strategies, media) within a specific intervention.

### Further suggestions

Finally, from jointly considering the EE and STEM literatures, two further suggestions for future research on early EE emerge. First, cross-disciplinary linkages may exist between EE and STEM education that may warrant simultaneous inclusion in future research (e.g., Huang et al. 2018). For example, although the focus of the entrepreneurship interventions was primarily on fostering direct entrepreneurial outcome variables (e.g., ESE), more general other skills such as communication skills or presentation skills were also being promoted. Therefore, the question arises whether other self-efficacies could be promoted, too. In particular, it would be interesting, both from an educational policy perspective as well as from a pedagogical point of view, whether it might be possible to promote different types of self-efficacy through tailor-made combinations of elements within one intervention. For example, in STEM education research, a few interventions focused on the simultaneous promotion of several STEM-related self-efficacies. For example, Falco and Summers (2017) and Huang and colleagues (2018) analyzed the development of STEM-related self-efficacies and self-efficacies belonging to other disciplines, that is, career-specific self-efficacy and ESE, respectively. Both studies reported positive results—even though in the case of Huang et al. (2018), they need to be viewed with some caution due to the absence of a control group and the lack of a pretest. This approach tentatively suggests that future studies may find it worthwhile to consider designing and evaluating interventions from a cross-disciplinary viewpoint (e.g., by promoting different self-efficacies or even using interdisciplinary theoretical frameworks). Furthermore, such cross-disciplinary research at the interface of STEM and entrepreneurship appears extremely relevant from a practical point of view, given major trends in the labor market towards an increasing importance of both STEM-related competencies and entrepreneurial competencies: The development of specific interventions, targeted at the simultaneous promotion of STEM SE and ESE may help prepare students better for their future professional lives. Thus, we suggest that future EE research should include cross-disciplinary approaches that consider STEM-related outcomes, thereby moving it forward as a design science.

It also implies that future research may seek to explore other ‘boxes’, too, that is, other possible complements to early EE. Research on (foreign) language learning (e.g., Raoofi et al. 2012) is a point in case: In recent years, studies have pointed to the important role of language in entrepreneurial contexts. Clarke and Cornelissen (2014, p 383) developed a generalized conceptual perspective “on the formative role of language in shaping the ideas of entrepreneurs and their attempts to gain a broader understanding and recognition […] from relevant stakeholders and resource providers.” Mastery of language in making their case thus appears to constitute a key skill of successful entrepreneurs. Other scholars have found that the use of, specifically, a foreign language affected individuals’ decision-making and behavior in relation to core aspects of entrepreneurship such as cooperation and competition (e.g., Urbig et al. 2016, 2020). And it appears that any such behavioral or decision-making related changes are prompted not so much by an individual’s objective language skills but rather by his or her subjective perception (e.g., Neeley 2013)—a finding that aligns with an emphasis on self-efficacy as a core antecedent of subsequent cognitive, attitudinal or behavioral outcome variables as adopted in this study. A joint consideration of EE and language-related education may also open up possibilities for further analyzing possible moderating effects, for example, of gender. Jones and Warhuus (2018), for example, used descriptions of entrepreneurship courses from universities in 21 countries to analyze the extent to which these descriptions used gendered language and to investigate how such language constructed gendered subjects. Based on finding strong evidence in favor of gendered language, they further explored implications for attracting students into courses as well as for implicit messages conveyed as to the sorts of persons who might succeed as entrepreneurs.

Finally, returning to the joint consideration of EE and STEM education research, both streams share a gap in terms of cross-country comparative studies. Five out of eight studies in EE and all 17 studies in STEM education research concentrated on the implementation of the focal intervention(s) in a single country. In EE research, the primary focus has been on Europe (five out of eight studies). STEM education research has mostly focused on the U.S. (ten out of 17 studies). Yet, pedagogical research has shown that culture may influence the utilization and effectiveness of different learning strategies (e.g., Joy and Kolb 2009; Woods et al. [in press]). Also, the worldwide PISA study has shown that characteristics of educational systems have far-reaching consequences for the development of various competencies (Reiss et al. 2019). Future studies in EE research might, thus, consider investigating the effectiveness of early educational interventions to foster ESE across different countries, thereby including different educational systems and cultures from a comparative perspective—and possibly uncovering the need to adapt interventions to local educational conditions for maximum effectiveness. A recent study by Woods and colleagues (in press) has vividly illustrated both the need for culture-specific pedagogy in EE as well as the extent and depth to which such adaptation may be desirable. By ‘reconstructing the entrepreneurship classroom through indigenizing pedagogy and learning’, Woods and colleagues (in press) pointed out the importance of acknowledging and appreciating in culture-sensitive EE the specificities of indigenous ways of knowing—as well as the possible benefits to EE in general from engaging with this knowledge, thereby enriching the overall body of knowledge in EE. In this respect, EE research is actually at an advantage in that the EE-oriented ‘BizWorld’ program (e.g., Rosendahl Huber et al. 2014) has meanwhile been implemented in 80 countries—a reach that none of the extant STEM interventions has, providing EE research with the opportunity to inspire STEM education research, in turn.

## Conclusion

In sum, research on early EE is still limited but growing (Rodrigues et al. 2012). Based on a systematic review of the literatures on fostering ESE through early EE and on promoting STEM-related self-efficacies through interventions in primary and secondary schools, we suggest that future research may benefit from (1) additionally considering an integrative approach including both design and evaluation of interventions, for example, by systematically varying the duration of interventions, specific learning strategies, teaching models, and media; (2) explicitly comparing country- and culture-specific influences; (3) accounting more comprehensively for moderating influences like learners’ gender, social background, or prior learning experiences and teachers’ characteristics; and (4) adopting a cross-disciplinary approach, especially at the intersection of entrepreneurship and STEM, in order to analyze the possibilities of an integrated approach to promoting different self-efficacies simultaneously.

## Data Availability

Not applicable.

## Footnotes

1 Future studies may find it instructive to conduct a similar survey among high-school students. During the Covid-19 pandemic (the time of this study), conducting such a survey in schools was prevented by the various restrictions.

2 Original quote in the native language (German): „Naturwissenschaften sind sehr abstrakte und damit anspruchsvolle Fächer, die je nach Klientel schwierig zu erreichen sind.“

3 Original quote in the native language (German): „Die Schüler kommen damit klar, wenn der Unterricht an lebensnahen Unternehmen erklärt wird, (…) ansonsten sind manche Dinge schwer zu erklären, zu abstrakt.“

4 Also, a few qualitative studies exist. However, due to the difficulty of directly comparing qualitative and quantitative studies, among others because of their diverging research foci and inherent methodological differences, we restrict this study to one type of study here, that is, quantitative studies. Both types of research methods have their own advantages and disadvantages. Qualitative studies tend to “generate rich, detailed, valid process data that usually leave the study participants’ perspectives intact” (Steckler et al. 1992, p. 1). Quantitative studies, in turn, tend to have advantages with respect to the generalizability of findings to larger populations (Steckler et al. 1992) and, thus, the possibility to derive future implications for other settings (Longva and Foss 2018). As we opted for the latter type of study, future research should find it worthwhile to conduct an analogous survey of qualitative studies—once there are a sufficient number of such studies available—and then compare the results with our findings.

5 Supplementary tables and figures are available from an online appendix through the Open Science Framework from: https://osf.io/z5nsc/?view_only=2c62b185a1474433b9c8cee766aee7f8.
